# Data set demonstrating an absence of touch effects on social orienting in adults

**DOI:** 10.1016/j.dib.2016.07.013

**Published:** 2016-07-16

**Authors:** Christy Reece, Richard Ebstein, Xiaoqin Cheng, Tabitha Ng, Annett Schirmer

**Affiliations:** a Department of Pediatrics and Reproductive Health, University of Adelaide, Australia; b Duke/NUS Graduate Medical School, Singapore; c LSI Neurobiology/Ageing Programme, National University of Singapore, Singapore

## Abstract

Forty-five women participated in a variant of the social orienting paradigm employed in “Maternal Touch Predicts Attentional Bias Towards Faces in Young Children” (Reece, in press) [1]. On a given trial, they saw a mathematical equation and indicated whether this equation was true or false. Equations were superimposed on face or house distractors. A female experimenter sat next to the participant. In separate blocks, she either rested her hand on the participants arm or refrained from touching. Performance was poorer on trials with face than house distractors. However, experimenter touch failed to modulate this effect. Here we present raw and analyzed data of this companion experiment.

## Specification Table

**Table:** 

Subject area	*Psychology*
More specific subject area	*Child Development*
Type of data	*Table*
How data was acquired	*E-Prime*® 2.0 Psychological Software
Data format	*Analyzed*
Experimental factors	*Images (Face/House) presented as background distractors in a visual object categorization task*
Experimental features	*Response times in ms collected for object classifications*
Data source location	*Singapore*
Data accessibility	*Data is with this article*

## Value of the data

• The paradigm employed by [1] is effective in producing a social orienting effect in adults.
• The data comprises mean response times for each participant and condition.
• The data presented here can be employed for individual statistical and meta-analysis.

## 1 Data

We present response time means computed for each participant and condition. In the table provided, the column “Image” refers to whether the distractor was a face or a house. The column “Touch” refers to whether the participant was or was not being touched by the experimenter. Response times are expressed in milliseconds.

## 2 Experimental design, materials and methods

We piloted the paradigm employed by [1] with 48 adult female participants. Three participants were excluded because they failed to follow instructions (*N*=2) or because they encountered a technical error (*N*=1). The remaining sample consisted of 45 females (mean age=21.07 years, *SD*=1.48) who completed this study in return for credits for an introductory level psychology course. Participants were predominantly Chinese (80%). The remaining sample consisted of Indian (7%) and Vietnamese (4%) participants, as well as one Bangladeshi, one Fillipino, one Burmese, and one undisclosed participant.

Rather than using the exact same procedure for children and adults, we introduced two variations in the adult pilot experiment. First, we used multiplication equations instead of geometrical shapes in order to avoid ceiling performance. Participants pressed one of two counterbalanced response keys to indicate whether an equation overlaid on a distractor was correct or incorrect (e.g., 2×2=5 would be incorrect). A second modification was that the experiment was divided into two counterbalanced blocks during one of which the experimenter rested her hand on the participant׳s forearm – a form of skin-to-skin contact that was deemed fairly appropriate between strangers. This modification was introduced to assess potential short-term touch effects on social orienting.

For statistical analysis, we trimmed correct trial reaction times to ±2*SD* and analyzed the resulting mean reaction times using a two-way analysis of variance (ANOVA) with Touch and Image as repeated measures factors. Participants had significantly longer reaction times during face trials (mean=1110.94 ms, *SD*=199.42) relative to house trials (mean=1088.52 ms, *SD*=196.45; *F*(1, 44)=4.64, *p<*.05) suggesting that they were more distracted by faces than houses. Importantly, the effect of Touch and its interaction with Face were non-significant (*p*s>.1). Thus, the pilot study replicated the well-established face bias in adults indicating that our paradigm is suitable for the study of social orienting. Additionally, the absence of differences between the touch and no-touch block suggested that immediate touch plays an insignificant role in affecting social orienting as measured with this procedure.

**Table:** 

Subject ID	Image	Touch	Response time
1	Face	Touch	808.6
1	Face	No_Touch	880.3
1	House	Touch	860.3
1	House	No_Touch	908.6
2	Face	Touch	1200.8
2	Face	No_Touch	1289.1
2	House	Touch	1109
2	House	No_Touch	1171.3
3	Face	Touch	1091.1
3	Face	No_Touch	912.6
3	House	Touch	1029.3
3	House	No_Touch	958.9
4	Face	Touch	1071.7
4	Face	No_Touch	1226.8
4	House	Touch	950
4	House	No_Touch	1192.7
5	Face	Touch	1324.2
5	Face	No_Touch	1389.1
5	House	Touch	1459.9
5	House	No_Touch	1402.9
6	Face	Touch	1128
6	Face	No_Touch	1023.5
6	House	Touch	1045
6	House	No_Touch	973.2
7	Face	Touch	1072.6
7	Face	No_Touch	1122.8
7	House	Touch	1042.6
7	House	No_Touch	1117
8	Face	Touch	1099
8	Face	No_Touch	1095.9
8	House	Touch	1066.5
8	House	No_Touch	1026.6
9	Face	Touch	1373
9	Face	No_Touch	1428.5
9	House	Touch	1366.1
9	House	No_Touch	1391.6
10	Face	Touch	1317.3
10	Face	No_Touch	1274.8
10	House	Touch	1221.6
10	House	No_Touch	1195
11	Face	Touch	905.4
11	Face	No_Touch	1065.4
11	House	Touch	875.2
11	House	No_Touch	1092.4
12	Face	Touch	1163.9
12	Face	No_Touch	1057.1
12	House	Touch	1067
12	House	No_Touch	935.2
13	Face	Touch	1075.2
13	Face	No_Touch	1057.4
13	House	Touch	1081
13	House	No_Touch	1174.1
14	Face	Touch	1096.5
14	Face	No_Touch	1098
14	House	Touch	1000
14	House	No_Touch	1000.8
15	Face	Touch	1184.9
15	Face	No_Touch	1109.6
15	House	Touch	1254.9
15	House	No_Touch	1111.3
16	Face	Touch	825.9
16	Face	No_Touch	756.1
16	House	Touch	755.4
16	House	No_Touch	771.3
17	Face	Touch	1090.8
17	Face	No_Touch	1035.7
17	House	Touch	1083.5
17	House	No_Touch	1131
18	Face	Touch	1084.5
18	Face	No_Touch	1087
18	House	Touch	1076.8
18	House	No_Touch	1065.8
19	Face	Touch	923.5
19	Face	No_Touch	1098.7
19	House	Touch	1058.6
19	House	No_Touch	1378.1
21	Face	Touch	849.4
21	Face	No_Touch	1061
21	House	Touch	944.4
21	House	No_Touch	1025.2
22	Face	Touch	1149.9
22	Face	No_Touch	1142.4
22	House	Touch	969
22	House	No_Touch	1088.2
23	Face	Touch	911.8
23	Face	No_Touch	822.4
23	House	Touch	1043.3
23	House	No_Touch	856.5
24	Face	Touch	909.9
24	Face	No_Touch	967.9
24	House	Touch	900.3
24	House	No_Touch	936.5
25	Face	Touch	849.3
25	Face	No_Touch	1040.3
25	House	Touch	845.3
25	House	No_Touch	975.8
26	Face	Touch	1376.9
26	Face	No_Touch	1590.6
26	House	Touch	1362.1
26	House	No_Touch	1551.9
27	Face	Touch	1277.3
27	Face	No_Touch	1271.9
27	House	Touch	1290.3
27	House	No_Touch	1223.8
28	Face	Touch	1223.6
28	Face	No_Touch	1127.1
28	House	Touch	1137.3
28	House	No_Touch	1019.9
29	Face	Touch	983.9
29	Face	No_Touch	1017.3
29	House	Touch	1009.3
29	House	No_Touch	1009.3
30	Face	Touch	1355.2
30	Face	No_Touch	1274.9
30	House	Touch	1283.3
30	House	No_Touch	1174.3
31	Face	Touch	899.3
31	Face	No_Touch	896.2
31	House	Touch	930.3
31	House	No_Touch	963.9
32	Face	Touch	1669.5
32	Face	No_Touch	1680.4
32	House	Touch	1775.6
32	House	No_Touch	1536.2
33	Face	Touch	1234.8
33	Face	No_Touch	1207.7
33	House	Touch	1353.5
33	House	No_Touch	1275.2
34	Face	Touch	1004
34	Face	No_Touch	1097.3
34	House	Touch	896.8
34	House	No_Touch	1163
35	Face	Touch	1014.7
35	Face	No_Touch	974.6
35	House	Touch	1000.3
35	House	No_Touch	917.5
37	Face	Touch	1305
37	Face	No_Touch	1123.1
37	House	Touch	1261.4
37	House	No_Touch	1064.8
38	Face	Touch	957.2
38	Face	No_Touch	1134.4
38	House	Touch	901.3
38	House	No_Touch	1079.1
39	Face	Touch	1120.7
39	Face	No_Touch	1270.3
39	House	Touch	1132.2
39	House	No_Touch	1145.1
40	Face	Touch	1082.6
40	Face	No_Touch	947.1
40	House	Touch	1056.4
40	House	No_Touch	875.7
41	Face	Touch	1467.1
41	Face	No_Touch	1449.6
41	House	Touch	1286.2
41	House	No_Touch	1255.9
42	Face	Touch	868
42	Face	No_Touch	936.5
42	House	Touch	894.3
42	House	No_Touch	995.3
43	Face	Touch	1027.4
43	Face	No_Touch	942.6
43	House	Touch	916.7
43	House	No_Touch	913.7
44	Face	Touch	1248.1
44	Face	No_Touch	1243.9
44	House	Touch	1130.2
44	House	No_Touch	1104.9
45	Face	Touch	857
45	Face	No_Touch	784.6
45	House	Touch	830.3
45	House	No_Touch	741.3
46	Face	Touch	839
46	Face	No_Touch	834.1
46	House	Touch	863.7
46	House	No_Touch	835.2
48	Face	Touch	1406.8
48	Face	No_Touch	1412.6
48	House	Touch	1216.1
48	House	No_Touch	1607.2

## References

[1] C.E. Reece, X. Cheng, A. Schirmer, Maternal touch predicts attentional bias towards faces in young children. Cognitive Development 39 (2016) 128-140.

