# Supplementary material for: Data set demonstrating an absence of touch effects on social orienting in adults
Source: Data Brief. 2016 Jul 16;8:904–9. doi: 10.1016/j.dib.2016.07.013 (PMC4961681; doi:10.1016/j.dib.2016.07.013)
Supplement: Supplementary file 1 — Supplementary material [file mmc1.doc]

I was unable to download a form from the website. There simply was no link visible to me.

I declare herewith that the authors of this manuscript have no conflict of interest.
